# Cementless unicompartmental knee replacement allows early return to normal activity

**DOI:** 10.1186/s12891-017-1883-8

**Published:** 2018-01-17

**Authors:** Benjamin Panzram, Ines Bertlich, Tobias Reiner, Tilman Walker, Sébastien Hagmann, Tobias Gotterbarm

**Affiliations:** Clinic of Orthopaedic and Trauma Surgery, University of Heidelberg, Schlierbacher Landstr. 200a, 69118 Heidelberg, Germany

**Keywords:** Cementless UKR, OUKR, Oxford medial, Physical activity, Sports, Return-to-activity, Knee arthroplasty

## Abstract

**Background:**

Physical activity and regular participation in recreational sports gain importance in patients’ lifestyle after knee arthroplasty. Cementless unicompartimental Knee replacement with the Oxford System has been introduced into clinical routine. Currently there is no data reporting on the physical activity, return to sports rate and quality of live after medial cementless Oxford Unicompartimental Knee Replacement (OUKR).

**Methods:**

This retrospective cohort study reports on the functional outcome of the first 27 consecutive patients (30 knees) that were consecutively treated with a cementless medial OUKR between 2007 and 2009 in our hospital. Physical activity and quality of life were measured using the Tegner-Score, the UCLA-Activity Score, the Schulthess Clinical Activity Questionnaire and the SF-36 Score. The patients’ satisfaction with the outcome was measured using a visual analogue scale.

**Results:**

Mean age at surgery was 62.5 years. Patients showed a rapid recovery with 17 out of 27 patients returning to sports within 3 months, 24 within 6 months after surgery. The Return-to-activity-rate was 100%. 10 out of 27 patients showed a high activity level (UCLA ≥7 points) with a mean postoperative UCLA-Score of 6.1 points.

**Conclusions:**

Patients recover rapidly after cementless OUKR with a return to sports rate of 100% and patients are able to participate in high impact sports disciplines.

## Background

As life expectancy is increasing and the incidence of osteoarthritis (OA) rises with age, there is a high demand of joint replacement for people of middle and advanced age. While the mean age of patients receiving UKR is 63.6 years, several studies show that patients perform high levels of activities up until after 70 years of age [1–3].

Therefore, physical functioning and participation in sports after surgery are important outcome measures of a successful joint replacement. To allow high levels of activity, UKR provides more physiological knee kinematics, a higher range of motion, a more natural perception of the knee, shorter hospital stay, faster recovery with a lower rate of complication when compared to Total Knee Replacement [4–6]. UKR has shown to have excellent long-term survival rates compared to TKR [7–9]. As the cemented UKR is a reliable treatment option for antero medial OA, its’ widespread use has been recommended for the elderly as well as for younger OA patients (e.g. patients <60 years) [4, 10–13].

For further improvement of the clinical outcome, implant survival and to eliminate complications associated with cementation, a cementless medial OUKR has been developed. Cementless fixation may offer several advantages compared to cemented OUKR. Reduced operation time, the absence of possible cement related tissue reactions, inefficient cementation which might influence the fixation or lead to early wear, clinical symptoms or even revision due to foreign bodies. Furthermore, radiographic radiolucent lines are less common suggesting a superior biological fixation [14]. Especially for patients at younger age with higher demands of sports activity these benefits seem to be desirable. However, cementless fixation is associated with a higher risk of intra and postoperative tibial plateau fractures and tibial valgus subsidence. Particularly an extended sagittal saw cut, a low bone density might lower the fracture load which may lead to tibial plateau fractures in combination with the firm impaction to achieve the desired press fit [15]. Valgus subsidence in cementless OUKR may be caused by extensive vertical saw cuts and or laterally implanted femoral components, causing impingement of the inlay against the medial tibial wall during flexion [16].

So far, there are promising short- to medium-term results published by the developing centres as well as registry data, indicating good clinical outcome and survival rates with a lower revision rate compared to the cemented version [17–20]. So far there are no published data reporting on the physical activity level after cementless OUKR. We therefore report in this study on the physical activity, return to sports rate and quality of life of our first consecutive 30 cementless medial OUKR.

## Methods

In this retrospective study we evaluated the first 27 patients (30 knees) who were treated consecutively with a cementless medial OUKR in our hospital. The study was assessed and approved by the ethics committee of the University of Heidelberg (S-546/2013). Surgery was performed by three experienced surgeons between 2007 and 2009 using the Oxford III System. The patient cohort was described in our previous work analysing the incidence of radiolucent lines in cementless fixation [21].

Patients were examined before surgery and at final follow-up. The level of physical activity before and after surgery was measured using Tegner and UCLA Activity Score. Detailed information about physical activity was obtained using the Schulthess Clinical Activity Questionnaire, which compares the state at follow-up with the last time point before the onset of OA symptoms. The SF-36 Score determines the self-perception of the patients’ quality of life, compared to a healthy cohort and a standard group suffering from osteoarthritis/ rheumatoid arthritis. The patient’s satisfaction with the operated knee was measured using a visual analogue scale (0–10).

The indication for operation in all cases was anteromedial osteoarthritis (OA) with intact lateral knee compartment. The anterior cruciate ligament (ACL) and the collateral ligaments were intact and the varus deformity was fully correctable manually. A flexion deformity >15° or previous osteotomy were contraindications for the procedure, while cartilage loss in the femoro-patellar joint, age and obesity were not considered as contraindications [22]. Indications were concordant with the recommendations by Goodfellow et al. [23]. After surgery, the patients followed a three-week rehabilitation scheme with full weight-bearing.

### Data analysis

SPSS Version 21 was applied to analyse the data. We used the Pearson’s Chi Square Test for categorial and ordinal variables. Comparison of Pre-and post-operative scores were performed utilising the Wilcoxon signed-rank test. To compare differences between two independent groups with ordinal or continuous variables we used the Mann-Whitney U test. *P*-values of 0.05 or smaller were considered as significant.

## Results

### Demographics and study group

The study group consisted of the first 27 consecutive patients (15 male, 12 female, 30 knees) that were consecutively treated with cementless OUKR in our institution. Patient age at surgery ranged from 49 to 76 years with a mean of 62.5 years. Mean follow-time after surgery was 60 months, raging from 47 to 69 months (SD 8.3).

No patient died during follow up. Overall 3 knees were excluded from the study. In one case the reason was a major deviation from the recommended surgical technique. One patient suffered a periprosthetic tibial fracture within the first month after initial operation with consecutive revision of the tibial component to a cemented version and ORIF. The third patient was excluded after total knee replacement following progressive OA of the lateral and the patellofemoral joint (PFJ). The remaining 27 knees (24 patients) were included in the clinical and functional assessment. We observed one reoperation due to dislocation of the mobile-bearing 21 months postoperative and consecutive exchange of the inlay to a thicker one. In one case OA of the PFJ resulted in additional patello femoral arthroplasty (PFA).

### Return to activity

Twenty-four out of 27 patients were physically active before surgery and all have returned to sports at final-follow up (see Table 1).

**Table 1:** Return to activity

		*After surgery*		Total
		Active (patients/knees)	Inactive (patients/knees)	(patients/knees)
*Before surgery*	Active	24/27	0/0	24/27
	Inactive	2/2	1/1	3/3
Total		26/29	1/1	27/30

**Fig. 1:**
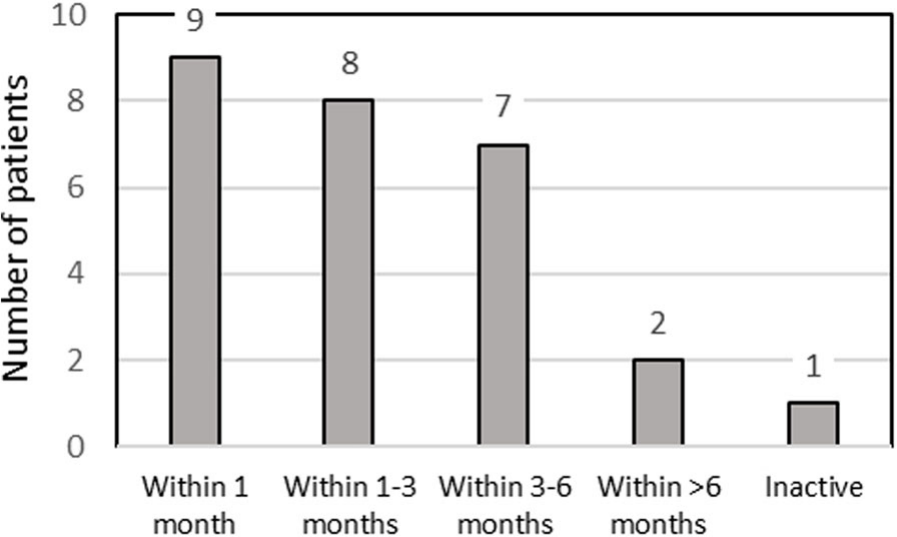
Return to activity

Seventeen patients (18 knees) returned to sports within 3 months after surgery (see Fig. 1). There were no age- and gender-related differences.

**Fig. 2:**
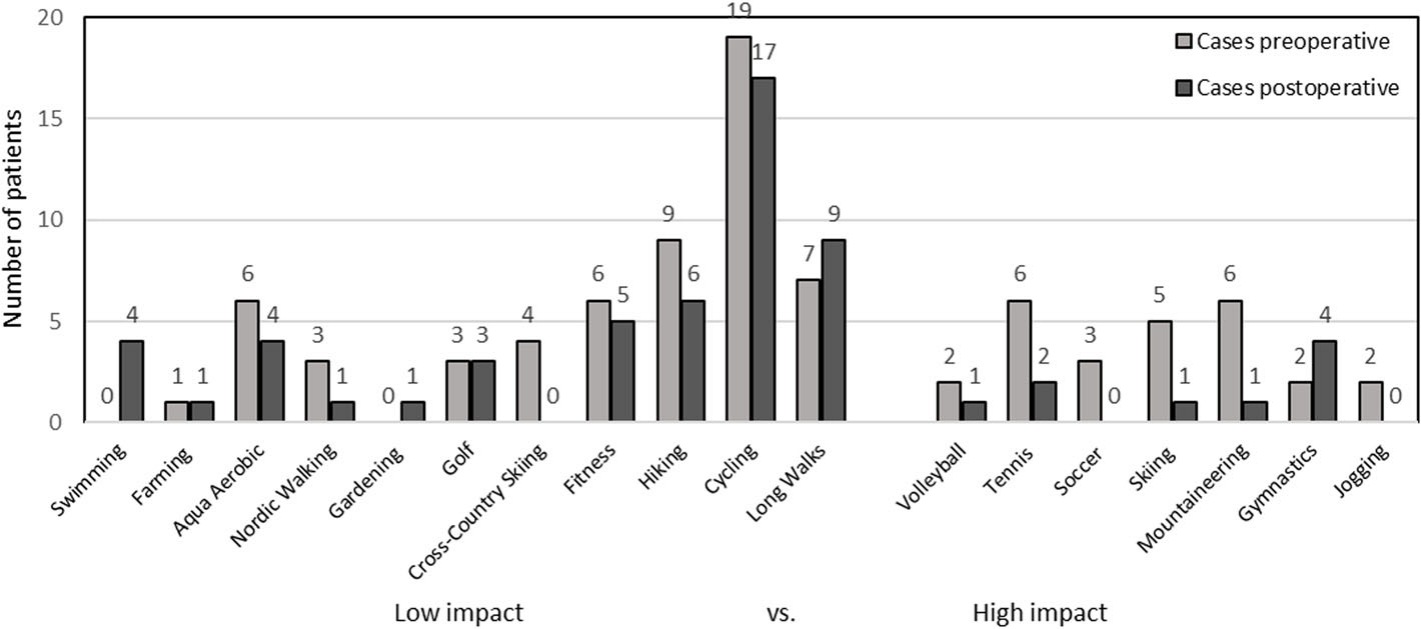
Sports and activities

Most popular activities before and after surgery were cycling, hiking and long walks (see Fig. 2). Altogether, 18 types of sport were performed pre- or postoperatively. There were 5 types of high-impact sports practised before the onset of OA symptoms as well as after surgery. There was a notable shift from giving up sports such as soccer or jogging and starting volleyball and mountaineering. The main cause for the change was “pain” (3 patients, 4 knees). The Change did not reach statistical significance. (*p* = 0.202).

### Amount of activities

There was no significant difference (*p* = 0.132) regarding the number of sports disciplines before the onset of OA symptoms and after surgery. During sports, 20 patients (22 knees) did not experience pain and 4 patients (4 knees) practised although feeling pain. One patient (one knee) did not participate in sports after surgery.

The quantitative assessment of sports participation was done using either the total number of patients that practised sports at least three times per week or the number of patients that practised at least 1 h per training session. There was no statistically significant difference comparing the state before OA onset and at follow-up in both parameters (*p* = 0.146).

While practicing sports, 19 patients (19 knees) felt excellent and did not report on any limitation or discomfort. Three patients (four knees) described a feeling of insecurity or fear of damaging the knee implant, three patients (three knees) felt they had a limit in the range of motion, and two patients each (two knees each) reported that they were not in a good physical condition or had a limited general flexibility.

### Scores and satisfaction

**Fig. 3:**
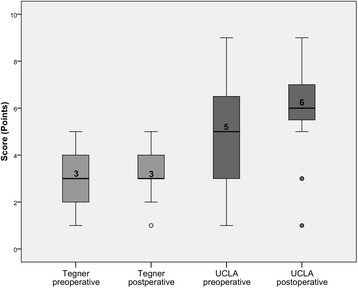
Tegner- and UCLA-Score before and after surgery

All 24 patients (27 knees) showed a significant improvement in both Tegner and UCLA-Scores after surgery (*p* = 0.042 each, see Fig. 3). UCLA–Score was 4.9 (SD 2.3) preoperatively and increased significantly to 6.1 after surgery (SD 1.8), with a mean change of 1.2 points (SD 1.8). Tegner Score improved by 0.5 points (SD 0.2) from 2.9 points before surgery (SD 1.4) to 3.4 points postoperatively (SD1.0). We classified the pre- and postoperative UCLA-Scores into three categories: ≥7 points: high activity levels, 4–6 points: moderate activity and ≤3 points: low activity [24]. At follow-up, 10 patients (11 knees) were highly active and 14 patients (12 knees) showed moderate activity. Three patients (4 knees) showed low activity levels.

**Fig. 4:**
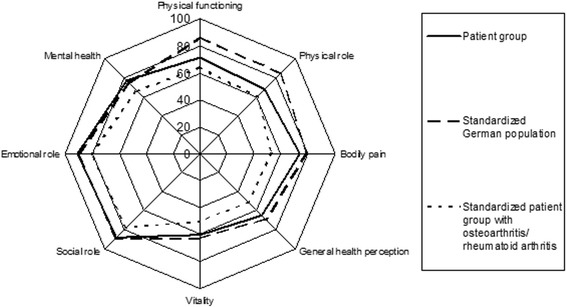
SF-36 Score

The SF-36 Score showed high score values in all patients with cementless OUKR at final follow-up (see Fig. 4). The Results compared to the two reference groups are shown in Fig. 4. Overall 23 patients were extremely and very satisfied with the outcome, 4 patients were satisfied.

## Discussion

This retrospective study assessed the physical activity and satisfaction in the first 27 consecutive patients (15 male, 12 female, 30 knees) that were treated with cementless OUKR in our institution between 2007 and 2009. Mean follow-up time was 60.0 months (47–69; SD 8.3) and mean age at surgery was 62.5 years (range 49–76).

Our main finding was that patients showed a high level of activity after cementless OUKR. Return-to-activity rate was 100% and the extent of activity did not differ from the time point before the onset of OA symptoms. The postoperative mean UCLA of 6.1 points and the predominant number of patients achieving 7 points or more in the UCLA-Score (10/26 patients) displayed that patients after cementless medial OUKR were able to reach a high level of impact sports.

In a meta-analysis by Witjes et al., the postoperative activity in 8 studies of patients receiving cemented UKR and 13 studies of patients receiving TKR was analysed. They found that the postoperative return-to-activity-rate as well as the level of activity were higher after cemented UKR than after TKR. They reported return-to-activity-rates between 75 - >100% in the UKR group and 36–89% in the TKR group. Our results indicate that cementless OUKR also allows patients high return- to-activity-rates compared to cemented UKR [25]. Although other authors presume that the effects of the learning curve, regarding the implantation of Oxford UKR, might affect the postoperative outcome and therefore the physical activity, our results show similar physical activity compared to cemented implantation [26, 27].

Our return-to-activity-rate showed higher values compared to studies about Oxford medial UKR, which range between 80.1% and 97% [3, 11, 28, 29]. In a retrospective study on the activity after cemented OUKR with a follow-up of 4.2 years, Pietschmann et al. reported a return-to-activity rate of 80.1% [3]. Possible reasons include the larger patient cohort and the high age of treated patients (131 patients with a mean age of 65.3 years compared to 27 patients with a mean age of 62.5 years). They split the patient collective into an active and an inactive group and found the active group to be significantly younger than the inactive group. They referred to the results of the “German Health Survey” 2006, which showed that activity decreased significantly in patients over 70 years of age. At five-year follow-up, the average patient in our study was 67.5 years old, compared to 69.5 years in Pietschmann’s study. Walker et al. reported a return-to-activity rate of 93% in patients of sixty years or younger after medial UKR. Almost two thirds reached postoperative UCLA-Scores >7 [11]. This matches the excellent outcome of our study with almost half of the collective being younger than sixty years. However, we did not detect a significant difference in the activity levels of patients older and younger than median age (data not shown).

In our study, patients did recover quickly after cementless OUKR, which supports literature findings [25, 30]. Price et al. showed minimal-invasive OUKR patients to recover twice as fast as standard incision UKR patients and three times as fast as TKR patients [31]. More than 60% of the patients in our study had picked up sports already during 3 months after surgery and 90% within the first 6 months.

Another finding of the present study is the high rate of patients without pain during sports (22 out of 26, 85%). Others have reported on the amount of patients being pain-free during activity ranging between 57% and 76% after cemented OUKR [11, 29]. A possible explanation for these different findings might be a shorter follow-up time of approximately 2 years compared to the five-year follow-up in our study.

In our study, UCLA-Score improved significantly from 4.9 points preoperatively to 6.1 points postoperatively (*p* = 0.042). This matches the findings of other studies: Fisher et al. reported an improvement from 4.2 to 6.5 points [28]. Generally, postoperative UCLA-Scores range from 6.1 to 6.8 points [11, 25, 32, 33]. Tegner-Score values are the only activity-related item that was published so far on cementless medial OUKR. In the randomized controlled trial, Pandit et al. compared cementless- with cemented OUKR. They reported an improvement from 1.9 points preoperatively to 3.1 points 2 years after implantation and 2.9 points at five-year follow-up in the cementless group. At 2 years follow-up, the Tegner-Score was significantly higher in the cementless group compared to the cemented group, but the difference did not persist until 5 years after surgery [19]. Tegner Score in our study was 2.9 points preoperatively and 3.4 points postoperatively at a mean follow up of 5 years, with a significant improvement (*p* = 0.042).

Concerning the extent, frequency and length of activities in our study, we found that there was no significant decrease after surgery compared to the time before the onset of OA symptoms. Our findings match the results of other authors assessing activity after cemented OUKR as well as activity after cementless Total Hip Arthroplasty [11, 34].

Sports were divided into high-impact (with high peak loads in the joints) and low-impact forms (with constant low joint loads). In contrast to other authors assessing postoperative activity after UKR, we did not find a significant increase or decrease in any type of sports after surgery. There are several authors reporting a significant decrease of high-impact sports after surgery, while low-impact sports tend to increase [2, 3, 11, 35]. The reason for this is might be attributed to multiple causes such as the surgeon’s recommendations, lack of function, feeling of insecurity, pain, comorbidities etc. Asked for their reasons to abandon high-impact sports, the majority of patients in a study by Walker et al. named “precaution” (59%) and “less motivation” (20%), while pain only followed fourth with 9% [30]. Although there is no final conclusion on the best type of sports for patients after joint replacement, there seems to be a general consensus from surgeons to discourage patients from high-impact sports such as soccer and tennis [36]. Supporting this position, there is indication that high-impact sports lead to high joint loads and can thereby increase implant wear followed by a higher rate of complications and revisions [36, 37]. Mobile-bearing devices such as the cementless OUKR are known to minimize wear due to the fully congruent mobile bearing [38, 39]. In accordance with these findings, although the postoperative level of activity was high, there were no revisions due to implant wear in this five-year follow-up study. Pietschmann et al. report that although they noticed a significant decrease in high-impact sports, they did not detect a correlation between high impact sports and complication. General activity is necessary to maintain cardiovascular fitness and bone density. There are several studies indicating that bone density depends on frequency of activity as well as on the imposed skeletal forces, indicating that impact up to a certain level has positive effects on bone density and should not generally be discouraged [40–42].

Assessing the quality of life, our patients reported excellent results. Regarding the physical dimensions, they accomplished higher values than the reference group suffering from OA/ rheumatoid arthritis. In the emotional-social domains of SF-36, they reached the same or better scores than the healthy reference population. Naal et al. compared the findings of their population to a matched reference group and they achieved significantly higher scores in every domain [2]. A possible explanation for the higher scores could be higher preoperative scores of their collective or the short time between surgery and questioning (18 months). Recently operated patients tend to remember their state before surgery more easily. Both studies showed high SF-36 Scores and were comparable with the findings of Walker et al., who investigated activity after cemented OUKR in patients ≤60 years of age [11].

In the present study, in accordance with literature, patients reported a high satisfaction rate with the outcome of the joint replacement [3, 11].

Superior radiological osseointegration and no cement associated complications seem to be a clear advantage of cementless fixation, especially desirable for young and active patients. However, possible early intra and postoperative complications associated with cementless OUKR like periprosthetic fractures and valgus subsidence might impair early return to sports and activity. Overall these complications are rare and appear to be rather influenced by mistakes in the surgical technique [15, 16].

Main limitation of this study is the small number of patients. We did not collect preoperative SF-36 data which makes the efficacy of cementless OUKR hard to compare to other author’s findings, but as we aimed to compare the postoperative quality of life with a healthy reference group, we think that the conclusions are not impaired.

Furthermore, although the scores and questionnaires used in our study (Tegner, UCLA, Schulthess Clinical Activity Questionnaire) are validated for the evaluation of physical activity, it is a difficult to quantify and compare the results as many parameters can only be answered using a free text, which makes subcategorization difficult. Activity cannot be reduced to one parameter only, thus comparison of many aspects of activity is necessary. However, patients’ subjective perceptions of sports capability may be an outcome measure that outweighs the supposed objective parameters of physical activity.

Another major weakness of this study besides the retrospective design might be selection bias since the patients were recruited within a 3-year time period (2007–2009) between the first and last inclusion.

The strength of this study is its detailed information at a mean follow-up of 5 years. Not only UCLA- and Tegner Score were measured, but also individual information about sports disciplines, frequency and length of activity. This is the first study to give detailed information about sports and activity after cementless medial OUKR. No patient died or was lost to follow-up.

## Conclusions

This study demonstrates that patients treated with cementless OUKR achieve high activity levels after surgery. Furthermore, patients seem to participate in the same sports activities than before onset of OA. Cementless OUKR allows fast recovery and a high return-to-activity rate. Quality of life was excellent compared to the healthy reference group.

## Abbreviations

ACL: Anterior Cruciate Ligament
OA: Osteoarthritis
OUKR: Oxford Unicompartimental Knee Replacement
PFA: Patello Femoral Arthroplasty
PFJ: Patello Femoral Joint
UKR: Unicompartimental Knee Replacement
